# Shortage in general practice despite the feminisation of the medical workforce: a seeming paradox? A cohort study

**DOI:** 10.1186/1472-6963-8-262

**Published:** 2008-12-17

**Authors:** Tanja Maiorova, Fred Stevens, Jouke van der Zee, Beppie Boode, Albert Scherpbier

**Affiliations:** 1 Institute of Medical Education, Faculty of Medicine, Maastricht University, The Netherlands; 2 Educational Development and Research, Faculty of Medicine, Maastricht University, The Netherlands; 3 NIVEL, Netherlands Institute of Health Services Research, Utrecht and Department of Care and Nursing, Maastricht University, The Netherlands; 4 Department of General Practice, Faculty of Medicine, Maastricht University, The Netherlands

## Abstract

**Background:**

Female medical students often prefer primary care specialties, while male students appear to be attracted to hospital specialties. Notwithstanding the steady feminisation of medicine, in many countries there are still difficulties in recruiting trainees for general practice. This seeming paradox raises the question on what specific role gender plays in a specialty choice. The authors looked at the (a) the role of gender in general practice specialty choice of Dutch medical students, (b) the decisive factors in career choice and relation of gender to these, and (c) differences in how male and female students are influenced by the GP clerkship.

**Methods:**

A cohort of 206 final year medical students at the Maastricht University, the Netherlands were asked to complete a questionnaire focusing on career preferences before and after a 12-week general practice clerkship and at graduation, a couple of months later.

**Results:**

Gender was significantly related to willingness to become a GP in bivariate analysis. Adding variables in multivariate analysis made this effect disappear. While females expressed overall higher preference for general practice than males, after the GP clerkship likelihood of choosing general practice increased with 38% among male and 22% among female students. After graduation, interest in general practice had dropped, mainly among females. Attitudes predicting a GP career choice were: extrinsic career motivation before the clerkship, and the content of GP work (patient contacts, treatments) and motivation to work with chronic and palliative patients after the clerkship.

**Conclusion:**

Gender 'as such' appeared not to be a distinctive predictor of specialty choice. It is students' attitudes towards GP work and preferred patient category that determine the career choice in general practice. However, more male students were positively influenced by the GP clerkship than female students. The motivating effect of the clerkship is not long lasting. Especially female graduates change their interest in favour of other specialties, which may explain why eventually few students choose general practice. It might be worthwhile to reinforce an initial preference for general practice by motivational guidance throughout the whole period of clerkships.

## Background

All over the world a steady feminisation of medicine is taking place [1-7]. Formerly male-dominated specialties are nowadays overrepresented by women [8]. In the Netherlands in 2008 65% of medical students were female [9]. Since 1970 the percentage of women doctors in the UK has risen by over 40% [5]. In the US in 2007 49% of medical students are female, compared to only 13% female medical students in 1970 [10,11]. This will have consequences for the future supply of physicians in different medical specialties, as gender is found to be one of the strongest demographic determinants of specialty choice [12]. According to a number of studies done in the UK, USA, Australia, Norway and the Netherlands, women show a strong preference for community-based careers, whereas men tend to prefer hospital-based ones [13-17].

Gender differences in medical specialty preferences have been explained from structural as well as from individual theories [18,19]. Seen from a structural perspective, a choice for a certain medical specialty depends for an important part on the acceptance of the work environment and conditions in which the career will take place. For example, a medical student who wants to specialize in a surgical specialty and to work in a hospital must be willing to accept hierarchy and long and irregular working hours. By contrast, doctors who wish to work more autonomously and to combine a career with time for their family will not be likely to invest in specialist training which involves much absence from home and family, frequent duties and long work days. In this regard, community-based specialties provide more stable structural opportunities to work part-time without shifts.

Individual-oriented explanations of specialty choice have as their starting point that interests in and attitudes towards specialties are in themselves strongly gender-based. For example, women value patient contacts more, while men usually score higher in biomedical orientation, academic interest, prestige en (high) income expectations.

There is also evidence that men are more likely to choose technical challenge, earning potential, prestige, while women consider work conditions, part-time work and parental leave ability more important [13]. In this light, high scores on biosocial orientation and avoidance of role strain correlates positively with interest in primary care and are typical for women [20].

Steady feminisation of medicine in combination with higher preference for part-time work will influence the workforce outcome, in particular in general practice, i.e. one would expect increasing numbers of medical students entering general practice. However, a peculiar paradox is that there are still difficulties in recruiting trainees for general practice [21]. While several factors may contribute to the current and predicted physician workforce problems in primary care, it remains worldwide a fact that few graduates intend to become a general practitioner [8,12,22-24]. This contradiction of feminisation on the one side and few graduates choosing for general practice on the other, raises the question on how gender is related to specialty choice and what other factors contribute to a certain specialty choice.

Past research identified several factors which influence the specialty choice in general practice. For example, early interest and experience in primary care significantly influences students' intentions of pursuing general practice as a future profession [14,25-28]. Medical students plan to enter general practice more frequently after having been exposed to primary care and after having done a clerkship in a general practice setting [25,29]. Clerkships are an important stage for students to put up their specialty preferences. In this paper we focus on the decision making process by analysing specialty preferences before and after the clerkship in general practice and investigate the role of gender in the motivation to become a general practitioner.

We sought to answer the following specific research questions:

(a) What is the role of gender in GP specialty preference?

(b) What are decisive factors in specialty preference and are these different for male and female students?

(c) Is there a gender difference in how medical students are influenced by the GP clerkship?

## Methods

### Participants

In 2002–2003, we surveyed students in the final year of the 6-year undergraduate medical curriculum of the University of Maastricht, the Netherlands. The clerkship in general practice (similar to family medicine in the U.S.) is compulsory and takes 12-weeks. Though the sequence of the clerkships is different for every student, the general practice clerkship always takes places in the final year.

Of the 206 registered students (55% women) participating in general practice clerkship, 184 students (56% women) completed questionnaire before and after the general practice clerkship. We asked the students to complete the questionnaire during group meetings at the start and at the end of the clerkship. In total 119 students (60% women) returned the postal questionnaire sent after graduation. Because some questionnaires were not fully completed, student's numbers in the analysis vary slightly.

### Instrument

New clerkship groups start every four weeks, the whole year through, between September and June. We asked the students to complete a questionnaire during group meetings at the start and at the end of the clerkship. Researchers distributed the questionnaires before the meeting and collected them afterwards. Students' consent was asked by the physicians responsible for the organization of the clerkships. At the end of the academic year, the students were sent a short postal questionnaire. The questionnaire, which was primarily based on previous studies mentioned above, contained questions about personal characteristics (age, gender, parents' occupation, previous study, and work experiences), general attitude towards a future physician career, general practice in particular, and likelihood to become a general practitioner. Factor analysis (varimax rotation) and scaling techniques were used to construct attitude variables from the questions about students' attitudes towards three main categories: (a) preferred patient category and type of work, (b) preferred work conditions in the future and (c) assessment of general practice as a profession. The items (5-point Likert scales), attitude variables (composed of sum scores), and reliabilities (Cronbach's alpha) are presented in table 1.

**Table 1:** Scales, items and reliabilities (alpha) concerning attitudes towards medicine and general practice in particular from medical students of the Maastricht University in 2002/03.

Scale	Items	Alpha
***Preferred patient category and type of work***:		
- Chronically ill patients and palliative care	Chronically ill patients	.72
	Geriatric patients	
	Palliative care	
	Long-term contacts with patients	
		
- Acute patients and technology-orientated work	Technical activities	.75
	Highly specialised work	
	Availability of personnel and equipment	
	Emergency care	
	Acute patients	
		
***Preferred work conditions***		
- Prestige orientation	Income	.70
	Career opportunities	
	High status	
		
- Controllable lifestyle	Part-time work	.76
	Regular working hours	
	Leisure time	
		
***Assessment of work in general practice***		
- Work intrinsic factors	Variety of patients and disorders	.68
	Contacts with family of patients	
	Individual treatments	
	Variety of work	
	Collaboration and communication with colleagues	
		
- Work extrinsic factors	High status	.63
	Income	
		
	Career opportunities	
- Work conditions	Working hours	.81
	Work load	

N = 175

The questionnaires at the start and at the end of the clerkship were identical. The postal questionnaire, sent after the students had qualified, contained a question on their ultimate specialty choice.

### Data analysis

We performed ANOVA (using SPSS) for gender, background characteristics, students' attitudes towards, and the likelihood of becoming a general practitioner (GP) before and after the general practice clerkship. Subsequently, we used multiple regression analyses to examine which factors predicted the perceived likelihood of becoming a GP before and after the clerkship. The likelihood of becoming a GP was the dependent continuous variable; background characteristics, preferred patient categories and type of work, preferences for future work conditions, and evaluation of becoming a GP were the independent variables. To compare the longitudinal changes in likelihood of becoming a GP between the three time points, we used one-way repeated-measures ANOVA. Because of variance or low frequencies, age (23–27 years old), marital status (2% married), and having children (1%) were left out of the analysis.

## Results

### Gender and determinants of career choice

Attitude variables related to career choice are differently related to gender (table 2). Female students were more likely to have considered an allied profession and to have work experience in patient care before studying medicine. Having participated in another health professions educational program did not differ for male and female students. Females were more attracted to work with chronic patients, palliative care and controllable lifestyle. Male students were more inclined to technology-oriented work, acute patients and prestige. Intrinsic aspects of a GP job were valued higher by women before the clerkship and were valued the same by men and women after the clerkship.

**Table 2:** Bivariate relationships between background characteristics, attitudes towards future work and general practice (mean score) and gender of medical students of the Maastricht University in 2002/03.

	Before the clerkship			After the clerkship		
	
	Mean (SD)			Mean (SD)		
	Male	Female	P value	Male	Female	P value
Likelihood to become a GP (1 = not likely, 5 = highly likely)	2.4(.7)	2.9(.9)	.00	2.7 (.9)	3.1 (1.1)	.003

*Background characteristics (0 = no,1 = yes)*						
Work experience in patient care	.3 (.4)	.6 (.4)	.00			
Other health care education	.3 (.4)	.4 (.4)	.084			
Having considered allied profession	.1 (.3)	.3 (.4)	.001			

*Preferred patients category and type of work (1 = not interested, 5 = interested)*						
Chronic patients and palliative care	3 (.7)	3.5 (.6)	.003	3(.7)	3.5(.6)	.00
Acute patient care and technology-orie oriented work	4 (.6)	3.6 (.6)	.001	4.2(.6)	3.8(.6)	.002

*Preferred work conditions (1 = not important, 5 = important)*						
Prestige orientation	2.65(.8)	2.15(.8)	.00	2.9(.7)	2.45(.8)	.00
Controllable lifestyle orientation	2.95(.9)	3.4(.8)	.001	2.85(.9)	3.5(.8)	.00

*Assessment of becoming a general practitioner (1 = not attractive, 5 = attractive)*						
Work intrinsic	3.8(.6)	4(.5)	.00	3.8(.6)	3.8(.6)	.125
Work extrinsic	2.6(.6)	2.6 (.5)	.399	3(.6)	3(.6)	.453
Work conditions	3.2(.8)	3.3(.8)	.618	3.5(.9)	3.5(.8)	.586

ANOVA was performed. P- values are two-sided. N = 175.

### Determinants of preference for general practice

Before and after the clerkship female gender was positively associated with a preference for general practice as a career (Table 3). This effect disappeared, however, when other independent variables were added to the regression analysis.

**Table 3:** Multiple regression analysis.

	Before clerkship		After clerkship	
Independent variables	Model 1	Model 2	Model 1	Model 2

Gender	.314**	.069	.222**	-.060
*Background characteristics*				
Having considered allied profession		.189**		.066
Physician parent		-.063		.074
Work experience in patient care		.033		-.007
Other health care education		.189**		.074
*Preferred patients category and type of work*				
Chronically ill patients and palliative care		.025		.148*
Acute patients and technology-oriented work		-.184*		-.260**
*Preferred work conditions*				
Prestige orientation		-.114		-.155*
Controllable lifestyle orientation		.087		.100
*Assessment of becoming a general practitioner*				
Work intrinsic		.121		.310**
Work extrinsic		.201**		.077
Work conditions		.018		.115
Adjusted R^2^	.097	.405	.043	.458

The dependent variable is the likelihood of becoming a general practitioner before and after the general practice clerkship among medical students of the Maastricht University in 2002/03. Independent variable values are standardized Beta. N = 175.

** p < 0.01; * p < .05

Before the clerkship, having considered an allied profession and having studied for another healthcare occupation were both positively related to a preference for general practice, as were positive attitudes towards the extrinsic job characteristics of general practice, such as status, income, and career opportunities. The preference of becoming a GP was lower among the students who had interest in acute patients and who were drawn to the technological aspects of medicine.

Comparing the predictors of general practice preference before and after the clerkships shows that a positive assessment of the extrinsic aspects of general practice was not related to a higher likelihood to become a GP afterwards. After the clerkship, motivation to work with chronically ill and palliative patients and positive assessment of the intrinsic aspects of general practice (treatments, contact with patients, and variety of work) became significant predictors of a higher likelihood of becoming a GP.

### Preference for general practice

**Figure 1:**
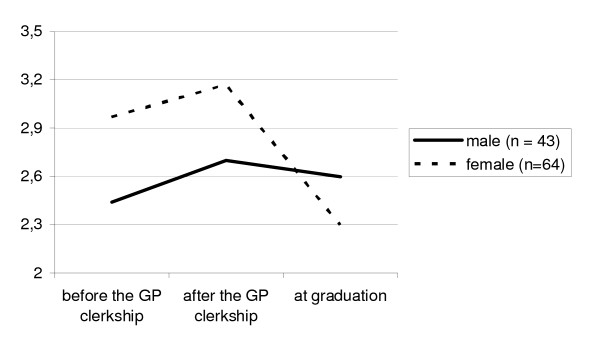
**Mean score of the likelihood of becoming a GP before and after a GP clerkship and after the graduation of medical students of The Maastricht University in 2002/03.** The mean score scale from 1 = unlikely to 5 = highly likely, N = 107.

The likelihood of pursuing a career in general practice before and after the clerkship and at graduation is represented on figure 1. After the clerkship, the overall preference for specialisation in general practice had risen among male and female students. The likelihood to become a GP increased among a higher percentage of male than female students (38% versus 22%; chi-square = 6.5, p < 0.05) although female students expressed a higher preference for general practice. By the time of graduation this preference had decreased again in both male and female students. One-way repeated-measures ANOVA was used to compare the mean scores before and after the GP clerkship and after the graduation separately for male and female students. Males F = 1.18, p = 0.92; females F = 9.42, p < 0.01. Remarkably, the overall likelihood of female graduates to become a GP became even lower than that of male graduates.

## Discussion

We investigated the effects of gender and a number of other factors on the perceived likelihood of planning a GP career before and after the clerkship in general practice. Previous studies have shown that gender is an important determinant of a specialty choice in general practice. In our multivariate analyses, however, we found that the relationship between gender and students' preferences did not hold after we included attitudinal variables. This raises the intriguing question of what the gender effect stands for. On the basis of the findings, we suggest that gender differences in selecting general practice as a specialty is not a clear-cut male-female distinction but instead reflects a combination of a number of factors, such as having a broader view of medicine, preferred type of work and patients and content of work. Regarding gender differences in medical specialty choices, two explanations were offered in the literature: individual and structural theory [7]. The question is how our findings fit into these theories.

The individual-oriented approach describes gender differences in terms of individual, gender-related interests and specialty preferences. In this study, interest in the *content of work *in general practice, measured by work-intrinsic factors such as kind of treatments, long term patient contacts, and variety of work, is the crucial factor when considering a career in this field. The finding that stimulation of students' interest in the type of problems faced in a certain specialty is the strongest motivational factor of the specialty choice, concurs with previous research [30]. It is important to note, however, that in our study the initial difference between male and female students in the assessment of work content in general practice has disappeared after the clerkship.

Our results further suggested that men were more interested in the technological and aspects of medicine and acute patients, whereas women were more interested in palliative care and chronically ill patients.

Although men and women had diverse primary interests in type of work and patients, this difference did not play an important role in the GP specialty preference. Similar conclusions were drawn from another study, where male and female residents were attracted to a particular specialty for similar reasons [24]. For both men and women an interest in the technological aspects of medicine and in acute patients was a particular negative predictor of a GP career preference. After the clerkship, an interest in working with chronically ill and palliative patients was a significant positive predictor of a becoming a GP. These findings are in line with previous research showing that generalists point out patient relationships as an important reason for selecting their specialty [31,24]. We conclude from this that the inner interest in the GP specialty does not discriminate between male and female students, and hence, the individualistic theory can only to some extent give an explanation of the backgrounds of the specialty choice in general practice.

The structural approach attempts to explain the gender difference in the light of work conditions and societal context. The disappearance of the impact of extrinsic factors (status, income, and career opportunities) after the clerkship in favour of work-intrinsic factors underscores the importance of inner interest in the GP specialty. Assessment of work conditions (work load and hours) in general practice did not predict the likelihood to become a GP either. Additionally, despite our expectations based on previous studies, controllable lifestyle options did not relate to a GP specialty preference.

The fact that individualistic theory only partially explains the difference between male and female students in our study, and that structural approach did not substantiate the results, asks for additional explanations [18,32]. First, these theories were applied some time ago and the interests of a new generation of male and female students may have become more alike. Students seem to identify more with the values of the preferred specialties rather than with values of the same gender [33]. In recent years women became more interested in traditionally "male' specialties, such as surgery [18,34,35]. Furthermore, our results show that although women are more inclined to enter general practice, a higher percentage of male than female students was motivated by the clerkship to become a GP. In other words, the preferences of men and women are likely to converge.

Another finding is that medical students ground their initial choices on the content of a subject matter. At the undergraduate stage work conditions seem less important to them. Probably, the question of a balance between work and private life is a question of later concern. There is evidence from earlier research that women were initially as likely as men to start a career in surgery and internal medicine [18]. However, later on, a high proportion of women did not complete their training because of difficulties of combining work and child care. They switched to other specialties. Our conclusion is that lifestyle options become more important on the later stage. This finding is not inconsistent with results of previous research where medical graduates have increasing preference for specialties with controllable lifestyle [35]. This could be due to the differences in the medical undergraduate education in U.S. and The Netherlands.

With regard to other factors affecting the choice for general practice, we found that having studied another, medicine related discipline had a positive predictive value. We surmise that these students have been able to develop a broader view on medicine and, therefore, are more likely to opt for a GP career. Students with "broad-based" undergraduate preparation frequently chose specialties with many doctor-patient interactions [36]. Thus, a broader view of medicine and health care, whether gained from earlier work experience or from studies other than medicine, increases the likelihood that a student will follow a career in general practice.

In this study, the factors we have explored seem to offer only a partial explanation for the perceived likelihood of becoming a general practitioner. Possible other determinants such as personality characteristics and social background may be included in future research.

We started this article with describing the paradox of the increasing influx of women and new generations of physicians in medicine on one hand, and the difficulties to recruit sufficient trainees for general practice on the other. Our study showed that men and women do not differ substantially in their interest in general practice. Flexible work conditions, which are mainly the preference of women, are not important at the undergraduate stage when initial specialty preferences are shaped. Hence, an increasing share of female students would not directly lead to the increase in the popularity of general practice.

The question, however, is what happens to all the potential trainees for general practice at graduation? Our results show that the positive effect of the clerkship was not long lasting, and relatively few students persisted in their initial plan to become a general practitioner after they had graduated. At graduation the interest in general practice had dramatically fallen amongst female students and became even lower than among male students. This drop may partly explain the inconsistency between the expectations of increasing interest in general practice care and low popularity of this specialty. Zinn and colleagues, in their study of students' primary care orientation, noted a decline in primary care interest among medical students between the fourth year and the residency [37]. Residents placed more value on technical aspects and had less interest in psychosocial issues. It might be that the positive experience during the clerkship in general practice is overshadowed by experiences from other clerkships in the final year. However, this explanation should be used cautiously, as the study did not show any relation between clerkships sequence and preference for general practice. While male graduates hold almost the same likelihood to become a GP, women seemed to favour other alternative specialties. In other words, it looks as if men are more firm in their GP decision than women, who might first want to try a hospital career.

Based on our findings, we suggest some implications for the practice. In order to stimulate students to choose general practice as a specialty, emphasis should be placed on the attractiveness of the content of work and type of patients in general practice. Other factors like working conditions and lifestyle considerations are less salient in shaping specialty preferences of undergraduate students.

Furthermore, seeing that the increased interest of students in general practice after the clerkship rotation was found to have diminished at graduation particularly among women, it seems advisable to take measures to sustain students' interest in general practice also after the clerkship, for instance by offering them experiences in general practice throughout the clinical phase of the curriculum until graduation. To support our findings, a similar survey could be replicated across more than one cohort of students.

## Conclusion

Predominantly individual interest in the content of GP work and preferred type of patients to work with determine the specialty choice in general practice, regardless of gender. Gender 'as such' appeared not to be a predictor of career choice. Male and female interests in medical specialty choice tend to converge. Work conditions do not play a role in the specialty preference at the undergraduate stage. Even though more females prefer to become a GP, males are influenced more by the clerkship than female students. However, the motivating effect of the clerkship is not long lasting. Mainly female graduates change their interest in favour of other specialties. This might explain why in spite of the rising number of female medical students general practice is still not a popular career choice among students. It might be worthwhile to reinforce an initial preference for general practice by motivational guidance throughout the whole period of clerkships. There is more longitudinal research needed to explore why students abandon the option to become a GP and what are the factors which play role in the definitive specialty choice of medical graduates.

## Competing interests

The authors declare that they have no competing interests.

## Authors' contributions

All authors designed the study and contributed to the development of the questionnaire. TM collected and analyzed the data, and drafted the manuscript. BB was responsible for data acquisition and contributed to the manuscript. AS, JvdZ, FS revised the different drafts critically for important intellectual content.

## Pre-publication history

The pre-publication history for this paper can be accessed here:


